# Predicting personalized cumulative live birth rate after a complete in vitro fertilization cycle: an analysis of 32,306 treatment cycles in China

**DOI:** 10.1186/s12958-024-01237-3

**Published:** 2024-06-07

**Authors:** Leizhen Xia, Shiyun Han, Jialv Huang, Yan Zhao, Lifeng Tian, Shanshan Zhang, Li Cai, Leixiang Xia, Hongbo Liu, Qiongfang Wu

**Affiliations:** 1 Reproductive Medicine Center, Jiangxi Maternal and Child Health Hospital Affiliated to Nanchang Medical College, Nanchang, China; 2 Jiangxi Key Laboratory of Reproductive Health, Nanchang, China; 3 Department of Health Statistics, School of Public Health, China Medical University, Shenyang, China; 4 Columbia College of Art and Science, the George Washington University, Washington, DC USA; 5 Department of Child Health, Jiangxi Maternal and Child Health Hospital Affiliated to Nanchang Medical College, Nanchang, China; 6 Department of Acupuncture, the Affiliated Hospital of Jiangxi University of Traditional Chinese Medicine, Nanchang, China

**Keywords:** In vitro fertilization, Cumulative live birth rate, Predictive model, Restricted cubic splines

## Abstract

**Background:**

The cumulative live birth rate (CLBR) has been regarded as a key measure of in vitro fertilization (IVF) success after a complete treatment cycle. Women undergoing IVF face great psychological pressure and financial burden. A predictive model to estimate CLBR is needed in clinical practice for patient counselling and shaping expectations.

**Methods:**

This retrospective study included 32,306 complete cycles derived from 29,023 couples undergoing IVF treatment from 2014 to 2020 at a university-affiliated fertility center in China. Three predictive models of CLBR were developed based on three phases of a complete cycle: pre-treatment, post-stimulation, and post-treatment. The non-linear relationship was treated with restricted cubic splines. Subjects from 2014 to 2018 were randomly divided into a training set and a test set at a ratio of 7:3 for model derivation and internal validation, while subjects from 2019 to 2020 were used for temporal validation.

**Results:**

Predictors of pre-treatment model included female age (non-linear relationship), antral follicle count (non-linear relationship), body mass index, number of previous IVF attempts, number of previous embryo transfer failure, type of infertility, tubal factor, male factor, and scarred uterus. Predictors of post-stimulation model included female age (non-linear relationship), number of oocytes retrieved (non-linear relationship), number of previous IVF attempts, number of previous embryo transfer failure, type of infertility, scarred uterus, stimulation protocol, as well as endometrial thickness, progesterone and luteinizing hormone on trigger day. Predictors of post-treatment model included female age (non-linear relationship), number of oocytes retrieved (non-linear relationship), cumulative Day-3 embryos live-birth capacity (non-linear relationship), number of previous IVF attempts, scarred uterus, stimulation protocol, as well as endometrial thickness, progesterone and luteinizing hormone on trigger day. The C index of the three models were 0.7559, 0.7744, and 0.8270, respectively. All models were well calibrated (*p* = 0.687, *p* = 0.468, *p* = 0.549). In internal validation, the C index of the three models were 0.7422, 0.7722, 0.8234, respectively; and the calibration P values were all greater than 0.05. In temporal validation, the C index were 0.7430, 0.7722, 0.8234 respectively; however, the calibration P values were less than 0.05.

**Conclusions:**

This study provides three IVF models to predict CLBR according to information from different treatment stage, and these models have been converted into an online calculator (https://h5.eheren.com/hcyc/pc/index.html#/home). Internal validation and temporal validation verified the good discrimination of the predictive models. However, temporal validation suggested low accuracy of the predictive models, which might be attributed to time-associated amelioration of IVF practice.

**Supplementary Information:**

The online version contains supplementary material available at 10.1186/s12958-024-01237-3.

## Introduction

In vitro fertilization (IVF) is the most common therapeutic option for couples with continuously unresolved fertility problems. It is estimated that the total number of IVF cycles conducted is over 1 million, with more than 400,000 babies born around the globe every year [1]. For patients who undergo treatment, live birth is the most crucial criterion to determine IVF success. Success rates of IVF are traditionally reported as live birth per embryo transfer (ET). With the increasing use of embryo freezing and thawing, it is essential to evaluate the cumulative live birth rate (CLBR) following multiple transfer cycles as the ultimate measure of success [2].

A precise predictive model of CLBR could help to achieve expected outcomes as much as possible for couples during assisted reproductive technology (ART) treatment, reduce the surgical risk and formulate appropriate individualized treatment for patients. Few studies have developed the predictive model to estimate the CLBR. McLernon DJ et al. developed multiple predictive models to estimate the chances of a live birth over multiple complete IVF cycles based on national data from the United States and the United Kingdom [3–5]. However, more scholars prefer the CLBR of a complete cycle, which can directly evaluate the efficiency of a single ovarian stimulation cycle and has more significance for clinical detail evaluation [6]. Existing models for CLBR after a complete IVF cycle were mainly based on the first IVF cycle and whole freeze-all IVF cycle [7–10].

Many IVF predictive models based on clinical outcomes have been developed in previous studies, and emerging patient and treatment characteristics have been proved to be important predictors [3, 11–13]. However, those models are rarely used in clinical practice. It could be blamed on the inconsistencies in the variables included and the poor predictive precision [14]. Thereinto, the precise relationship between variables and IVF outcomes is a key impediment to improve predictive accuracy. Several predictors like female age, antral follicle count (AFC) and oocyte number were considered to be non-linearly associated with the chance of live birth [15–17]. However, there are rare studies to exploit the nonlinearity in the models. Churpek et al. reported that using restricted cubic spline allows for predicting nonlinearity with higher accuracy than traditional logistic models in the context of critical care medicine [18]. Therefore, it is a necessary attempt to apply non-linearity in the IVF predictive models.

Embryo quality is another essential factor in IVF treatment, associated with implantation potential and live birth [19]. After completion of in vitro culture, embryologists assessed each embryo through visual inspection of morphological features [20, 21]. Following fresh transfer, supernumerary embryos with acceptable implantation potential were cryopreserved for subsequent frozen embryo transfer (FET) cycles to improve CLBR. However, given the adjustment of the transfer strategies in different cycles, it could be difficult for researchers to consider the effect that embryo quality poses on the CLBR in ART treatment [22, 23]. Current IVF predictive models about CLBR are lack of comprehensive consideration of all embryos in a stimulation cycle. We believe that to establish an available CLBR model with high precision, it is indispensable to include embryo characteristics. However, how to consider the predictive value of all embryo in a complete cycle for the CLBR becomes an urgent problem.

In this study, we aimed to develop a set of predictive models to assess the predicted cumulative probability of live birth. The first was a pre-treatment model for predicting the cumulative chance before couples receiving treatment; the second was a post-stimulation model to revise predictions after oocyte retrieval; and the last was a post-treatment model that comprehensively included information of all implantable embryos to predict success.

## Materials and methods

### Study design and participants

**Fig. 1:**
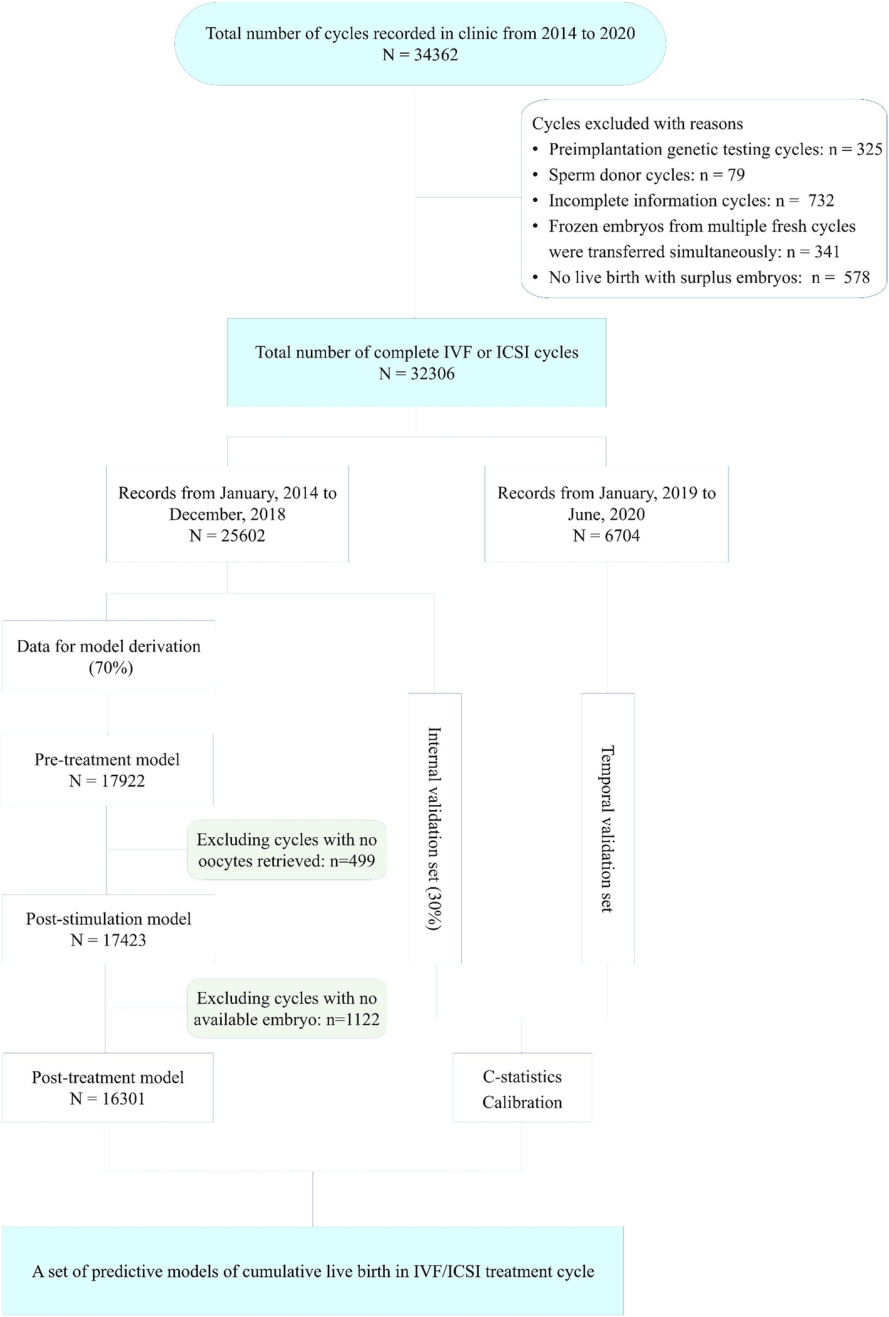
Flowchart of selection for the study

Between January 1, 2014 and June 30, 2020, we conducted a retrospective analysis of women undergoing IVF or intracytoplasmic sperm injection (ICSI) treatment at the Reproductive Medicine Center of Jiangxi Maternal and Child Health Hospital Affiliated to Nanchang Medical College. A total of 32,306 treatment cycles derived from 29,023 couples were included. All data of the subjects were retrieved from the electronic medical records of our center. The study protocol was approved by the Reproductive Medicine Ethics Committee of Jiangxi Maternal and Child Health Hospital (SZYX-202,306). The inclusion and exclusion criteria and study design are shown in Fig. 1.

### IVF/ICSI treatment procedure

Before commencing IVF treatment, couples underwent standard infertility assessments, including serum test and transvaginal ultrasound (TVUS). Ovarian stimulation was performed using recombinant follicle stimulating hormone and/or urinary human menopausal gonadotropins (rFSH/hMG). The main stimulation regimens included follicular phase gonadotropin-releasing hormone (GnRH) agonist protocol [24], luteal phase GnRH agonist protocol, and GnRH antagonist protocol. Follicular development was monitored by ultrasonography, and final oocyte maturation was induced by human chorionic gonadotrophin (hCG) when at least three follicles of 18 mm in diameter were observed. TVUS-guided follicular aspirations were performed 36 h after triggering.

Fertilization was carried out in vitro by either conventional IVF or ICSI depending on semen parameters. Embryos were cultured in sequential medium, with incubation conditions set at 6% CO_2_, 5% O_2_, and 37.0 °C. Recorded morphological characteristics included fragmentation, cell number and symmetry of all day 3 embryos with implantation potential.

Fresh embryo transfers were carried out under ultrasound guidance on day 3, day 5 or day 6. Supernumerary embryos were cryopreserved. The vitrification procedure was performed using the Cryo Bio System (France). Subsequent frozen-thawed transfer was performed through a natural cycle with hCG or through an artificial cycle with or without GnRH agonist pre-treatment [25]. The number of embryos transferred varied from one to two based on patient features.

### Main outcome measures

The main outcome was the CLBR of a complete IVF treatment cycle. The birth of at least one live-born baby per initiated cycle was defined as a live birth, and all the other adverse outcomes were classified as no live birth. A complete cycle included a fresh embryo transfer and associated frozen embryo transfers resulting from a single episode of ovarian stimulation.

### Statistical analysis

We developed three predictive models divided by three consecutive stages of a complete cycle, namely pre-treatment, post-stimulation and post-treatment.

#### Pre-treatment model

For a couple ready to embark on IVF treatment, we estimated the CLBR using the couple’s baseline characteristics and fertility check-up.

#### Post-stimulation model

For a couple who finished the initiated cycle and successfully retrieved oocytes (the number of oocytes > 0), we included stimulation treatment characteristics along with the features from the pre-treatment model.

#### Post-treatment model

For a couple who obtained implantable cleavage embryos after embryo culture, we assessed the CLBR based on the characteristics of post-stimulation model and cumulative Day-3 embryos live birth capacity. The cumulative Day-3 embryos live birth capacity indicates the potential for live births of all Day-3 embryos obtained from a single cycle. Specifically, we collected data about embryos morphology and outcomes of fresh single Day-3 embryo transfer from all complete cycles (*n* = 2594). We chose cell number, fragmentation and symmetry of Day-3 embryo as explanatory variables to develop logistic regression model of live birth probabilities. Then we imported relative data of all Day-3 embryos in every eligible complete cycle, calculated cumulative Day-3 embryos live-birth capacity of all cycles, and treated it as a new variable added to post-treatment model. The model information and calculation formula were shown in Supplemental text 1.

Continuous data were presented as the mean value ± standard deviation (SD) or median with interquartile range, and categorical data were described as the number of cases and percentages. Comparisons between the live birth and non-live birth groups were determined using Student’s t-test for continuous variables and Chi-squared test for categorical variables. To detect any possible linear or non-linear correlation between female age, AFC, number of oocytes retrieved, cumulative Day-3 embryos live birth capacity and the CLBR and to allow for a flexible interpretation of the relationships, continuous changes in these variables were assessed through restricted cubic splines (RCSs) [26]. We put 3, 4, 4, 3 cut-off points for female age, AFC, number of oocytes retrieved and Cumulative Day-3 embryos live birth capacity as the knots, respectively. Other continuous variables, including female body mass index (BMI) (< 18.5, 18.5 ~ 23.9, 24.0 ~ 27.9, ≥ 28 kg/m^2^), duration of infertility (< 2, 2 ~ 5, > 5 years), abortion number (0, 1, 2, > 2), number of previous ART treatments (0, 1, 2, > 2), number of previous ET failure (0, 1, 2, > 2), endometrial thickness (< 7, ≥ 7 mm), estradiol (E2) (< 1049, 1049 ~ 1795, 1796 ~ 2751, > 2751 pg/mL), progesterone (P) (< 0.47, 0.47 ~ 0.68, 0.69 ~ 0.97, > 0.97 ng/mL), and luteinizing hormone (LH) (< 0.65, 0.65 ~ 1.06, 1.07 ~ 1.96, > 1.96 mIU/mL) on trigger day were transformed into categorical variables before modeling.

We fitted multivariable logistic regression models to predict live birth through a complete cycle of IVF. Stepwise variable selection was used to remove uninformative variables in regression models. In short, starting from the full model performance, we iteratively remove one feature from the model while including last removed feature to the model, and assert whether the model has improved according to the akaike information ccriteria (AIC). The procedure is iterative and continues until there is no change in the AIC.

All eligible subjects were split by time. We used subjects in the first phase (January 2014–December 2018) for model derivation and internal validation, by randomly dividing into a training set and a test set at a ratio of 7:3. Subjects in the second phase (January 2019–June 2020) were used for temporal validation. When the sample size is very large, this approach has been shown to be methodologically more rigorous than a simple random split of the dataset [27]. The performance of the models was evaluated by means of C index and calibration. The C index, equivalent to the area under the receiver operating characteristic (ROC) curve, assesses the model’s discriminative capacity. Calibration refers to the level of agreement between the estimated and observed probabilities of a given event. Calibration was assessed by means of Hosmer-Lemeshow test. All analyses were performed with the use of the statistical software R software version 4.0.2 (http://www.R-project.org/). P-value < 0.05 was considered statistically significant for all analyses.

## Results

After exclusion, the study included 32,306 complete cycles (defined as all fresh and frozen embryo transfers resulting from one episode of ovarian stimulation) derived from 29,023 couples undergoing IVF treatment. Table 1 summarizes the distribution of the detailed characteristics and cycle information of the couples. Among all eligible cycles, 31,436 (97.3%) cycles had oocytes retrieved after ovarian stimulation, 29,397 (90.9%) obtained usable embryos after in vitro culture, and 18,758 (58.1%) ended with live birth after transfer.

**Table 1:** Characteristics of women and their treatment at a complete cycle

Characteristics	Pre-treatment (*n* = 32,306)	Post-stimulation (*n* = 31,436)	Post-treatment (*n* = 29,397)
**Baseline characteristics**			
Female age (y), Median (interquartile range)	31 (8)	30 (8)	30 (8)
Antral follicle count, Median (interquartile range)	11 (9)	11 (9)	12 (9)
Female BMI (kg/m^2^), Mean ± SD	21.97 ± 3.03	21.95 ± 3.02	21.94 ± 3.02
Duration of infertility (y), Mean ± SD	4.51 ± 3.52	4.48 ± 3.49	3.83 ± 3.41
No. of abortion, Mean ± SD	0.60 ± 0.97	0.60 ± 0.96	0.60 ± 0.93
No. of previous IVF attempts, Mean ± SD	1.36 ± 0.81	1.34 ± 0.78	1.31 ± 0.71
No. of previous ET failure, Mean ± SD	0.22 ± 0.61	0.21 ± 0.61	0.21 ± 0.60
Type of infertility, n (%)			
Primary infertility	13,695 (42.39)	13,346 (42.45)	12,409 (42.21)
Secondary infertility	18,611 (57.61)	18,090 (57.55)	16,988 (57.79)
Infertility diagnosis, n (%)			
Tubal factor	23,063 (71.39)	22,518 (71.63)	21,181 (72.05)
Male factor	8714 (26.97)	8500 (27.04)	7964 (27.09)
Ovulatory disorder	4636 (14.35)	4545 (14.46)	4349 (14.79)
Endometriosis	2224 (6.88)	2156 (6.86)	1968 (6.69)
PCOS	3017 (9.34)	2979 (9.48)	2917 (9.92)
Intrauterine adhesion, n (%)	5034 (15.58)	4908 (15.61)	4615 (15.7)
Scarred uterus, n (%)	3637 (11.26)	3534 (11.24)	3301 (11.23)
**Ovarian stimulation characteristics**			
Stimulation protocol, n (%)			
Follicular phase GnRH agonist protocol	-	23,472 (74.67)	22,758 (77.42)
Luteal phase GnRH agonist protocol	-	1894 (6.02)	1801 (6.13)
GnRH antagonist protocol	-	2861 (9.10)	2585 (8.79)
Others	-	3209 (10.21)	2253 (7.66)
Endometrial thickness on trigger day (mm), Mean ± SD	-	10.59 ± 2.67	10.68 ± 2.63
E2 level on trigger day (pg/mL), Mean ± SD	-	2113.38 ± 1433.09	2186.96 ± 1421.79
P level on trigger day (ng/mL), Mean ± SD	-	0.78 ± 0.58	0.78 ± 0.54
LH on trigger day (IU/L), Mean ± SD	-	1.68 ± 2.29	1.53 ± 1.92
Types of trigger, n (%)			
hCG	-	28,416 (90.39)	27,243 (92.67)
GnRH agonist	-	1562 (4.97)	1058 (3.60)
hCG + GnRH agonist	-	1458 (4.64)	1096 (3.73)
No. of oocytes retrieved, Median (interquartile range)	-	10 (9)	11 (8)
**Embryo transfer characteristics**			
Fertilization method, n (%)			
IVF	-	-	21,964 (74.72)
ICSI	-	-	6034 (20.53)
IVF + ICSI	-	-	1399 (4.76)
Cumulative Day-3 embryos live birth capacity, Median (interquartile range)	-	-	1.49 (1.66)

*BMI *Body mass index, *IVF *In vitro fertilization, *ET *Embryo transfer, *PCOS *Polycystic ovary syndrome, *GnRH* Gonadotropin-releasing hormone, *E2* Estradiol, *P* Progesterone, *LH* Luteinizing hormone, *hCG* Human chorionic gonadotrophin, *ICSI* Intracytoplasmic sperm injection

### Prediction of CLBR by pre-treatment model

**Fig. 2:**
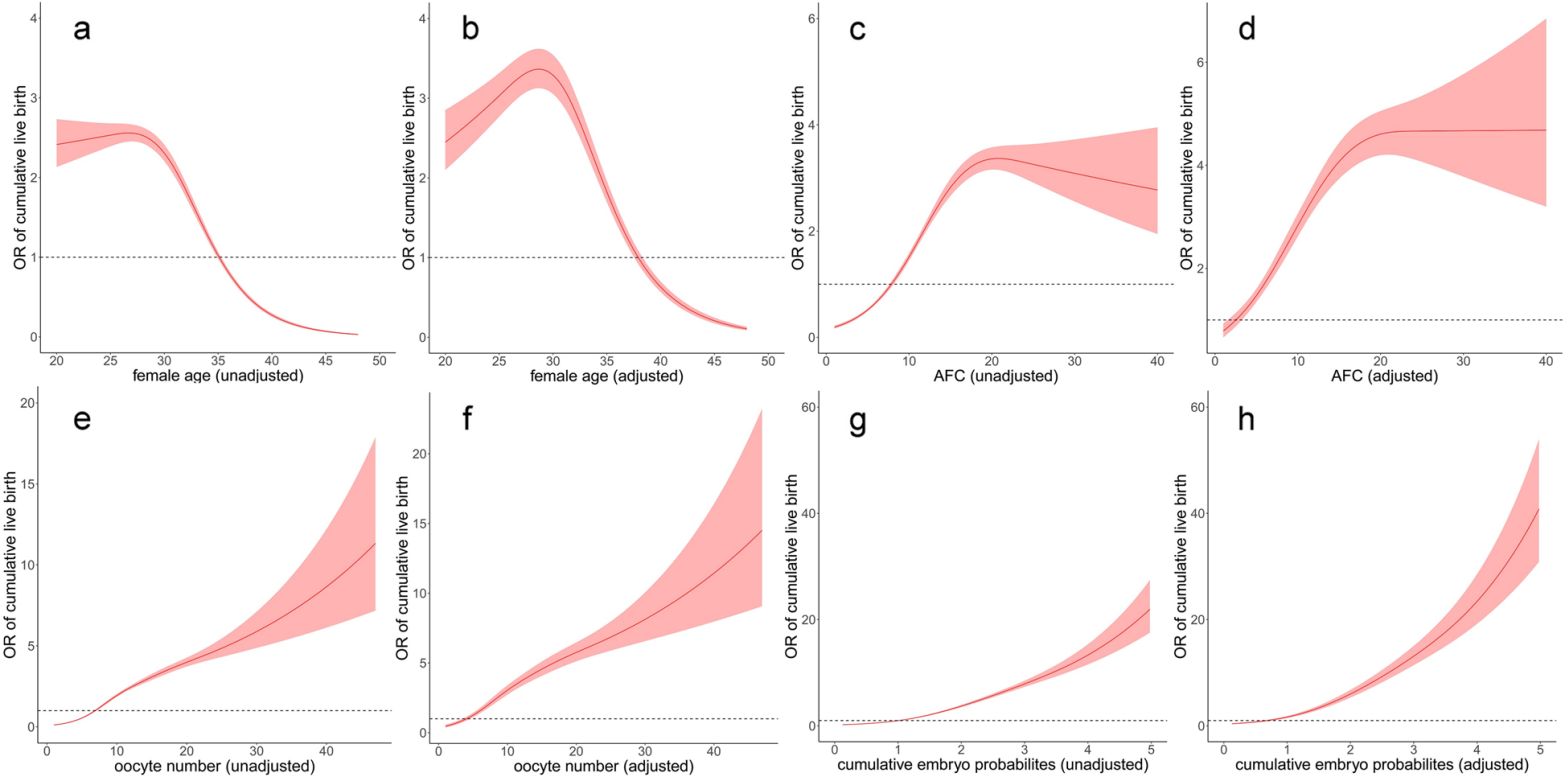
Non-linear association between predictors and cumulative live birth rate. Restricted cubic splines of the unadjusted and adjusted odds ratios of the cumulative live birth rate with female age (**a**, **b**), antral follicle count (**c**, **d**), number of oocytes retrieved (**e**, **f**), cumulative Day-3 embryos live birth capacity (**g**, **h**)

**Fig. 3:**
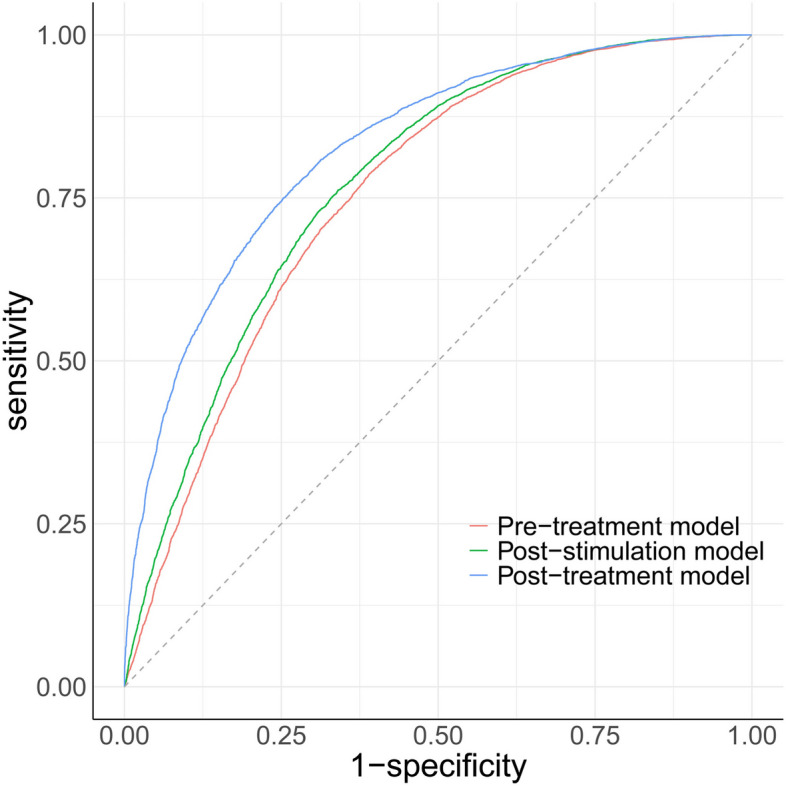
The area under the ROC curve of three predictive models

**Fig. 4:**
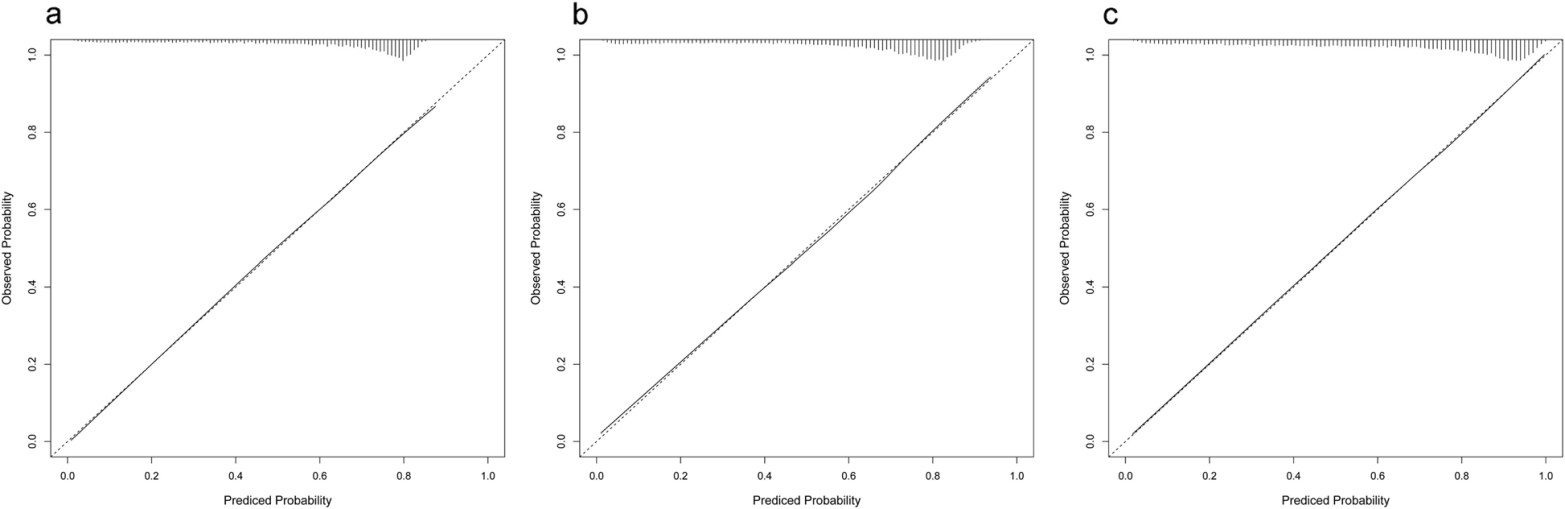
Calibration plots showing the observed cumulative live birth rate (95% CI) from the training dataset and the predicted cumulative live birth rate over the pre-treatment model (**a**), post-stimulation model (**b**), and post-treatment model (**c**)

A total of 17,922 cycles was included for this model. Univariable analysis showed that all baseline patient characteristics had statistically significant associations with CLBR, except for intrauterine adhesion (Supplemental Table 1). After multivariable logistic regression modeling with stepwise variable selection, the predictors of CLBR were female age, AFC, BMI, number of previous IVF attempts and ET failure, type of infertility, tubal factor, male factor, and scarred uterus (Table 2). The RCS (Fig. 2a-d) illustrated the relations between continuous changes of female age, AFC and changes in odds ratios (ORs) for the CLBR. As shown in Fig. 2 (a), there was a non-linear correlation between the change of unadjusted female age and ORs with an inverted S-shape curve. At the age of 26 ~ 27, the unadjusted OR was up to 2.559 (95%CI: 2.451–2.671) and then became a continuous downward trend. After adjusting for other confounding factors in logistic model, the non-linear relationship between female age and ORs of CLBR still remained (Fig. 2b). For AFC, the effect on unadjusted and adjusted ORs of live birth also shown a non-linear relationship with S-shape curves (Fig. 2c, d). With the increase of AFC, the effect turned to be positive (OR: 1.082, 95%CI: 1.026–1.142) until 8 ~ 9, reached the highest point (OR: 3.367, 95%CI: 3.153–3.595) at about 21, and began to descend afterward. Other covariates also made effects on CLBR. Increasing number of previous IVF attempts reduced the probabilities of live birth gradually (1, 2, > 2 vs. 0; adjusted OR: 0.389, 0.284 − 0.144), while the increment of previous ET failure improved the odds (1, 2, > 2 vs. 0; adjusted OR: 1.566, 2.308–2.696). Couples with a diagnosis of tubal or male factor infertility had slightly higher CLBR, with the ORs of 1.126 (1.041–1.218) and 1.147 (1.062–1.240), respectively. Women with vaginal sonograph of scarred uterus had reduced odds (OR: 0.803, 95%CI: 0.716-0.900). Finally, the pre-treatment model of cumulative live birth was established, the area under the ROC curve was 0.7559 (Fig. 3, pre-treatment model), and the model calibrated well (*p* = 0.687, Hosmer-Lemeshow test). The calibration curve was shown in Fig. 4 (a).

**Table 2:** Effect of each predictor on cumulative live birth rate of ART treatment adjusted for patient characteristics (Pre-treatment model)

Predictors	Coefficient	Odds ratio	95% CI	*P* value
Intercept	-1.826	0.161	0.097~0.266	<0.001
Female age	0.043	1.044	1.026~1.061	<0.001
Female age^a^	-0.206	0.814	0.795~0.832	<0.001
Antral follicle count	0.158	1.171	1.134~1.208	<0.001
Antral follicle count^a^	-0.180	0.836	0.716~0.974	0.022
Antral follicle count^b^	0.182	1.200	0.830~1.738	0.333
Female BMI, (vs <18.5)				
18.5~23.9	0.065	1.067	0.954~1.193	0.255
24.0~27.9	-0.035	0.965	0.846~1.100	0.596
>28.0	-0.212	0.809	0.664~0.986	0.035
No. of previous IVF attempts, (vs 0)				
1	-0.944	0.389	0.342~0.442	<0.001
2	-1.258	0.284	0.229~0.351	<0.001
>2	-1.936	0.144	0.104~0.198	<0.001
No. of previous ET failure, (vs 0)				
1	0.449	1.566	1.341~1.831	<0.001
2	0.836	2.308	1.832~2.912	<0.001
>2	0.992	2.696	1.906~3.818	<0.001
Type of infertility, primary infertility vs. secondary infertility	0.148	1.160	1.075~1.251	<0.001
Tubal factor, (yes vs no)	0.119	1.126	1.041~1.218	0.003
Male factor, (yes vs no)	0.137	1.147	1.062~1.240	<0.001
Scarred uterus, (yes vs no)	-0.220	0.803	0.716~0.900	<0.001

*BMI* Body mass index, *IVF* In vitro fertilization, *ET* Embryo transfer

^a^The binomial of restricted cubic splines

^b^The trinomial of restricted cubic splines

### Prediction of CLBR by post-stimulation model

To predict the CLBR in couples who successfully retrieved oocytes from the initiated cycles, we remained 17,423 eligible cycles and developed the post-stimulation model after adding new information of ovarian stimulation. In univariable analysis, the diagnoses of tubal factor and intrauterine adhesion had no insignificant associations with live birth (Supplemental Table 2). After stepwise selection from multivariable logistic regression model, a total of 10 predictors remained, as shown in Table 3. Compared to follicular phase GnRH agonist protocol, choosing luteal phase GnRH agonist, GnRH antagonist and other protocols reduced the odds of live birth by 30.4%, 24.7%, 39.4%, respectively. On the hCG trigger day, women with endometrial thickness ≥ 7 mm had mildly higher CLBR than those whose thickness was < 7 mm (OR:1.354, 95%CI: 1.152–1.592). Women with peak LH level of > 1.07 or > 1.96 mIU/mL had 13.5% or 23.5% improved live birth odds than those of < 0.65 mIU/mL, while higher P level reduced the odds (> 0.97 v < 0.47 ng/mL; OR:0.814, 0.731–0.907). RCS indicated the positive relationship between the oocyte number and the ORs of CLBR with four knots (Fig. 2e, f; *p* value for nonlinearity < 0.001). In multivariable adjustments, the odds of live birth turned to be significant until 4–5 (OR:1.172, 95%CI: 1.030–1.334), and the increasing trend became relatively steep after that point. The effects of other factors were similar to those appeared in the pre-treatment model. Finally, the post-stimulation model of cumulative live birth was established, the area under the ROC curve was 0.7744 (Fig. 3, post-stimulation model), and the model calibrated well (*p* = 0.468, Hosmer-Lemeshow test). The calibration curve was shown in Fig. 4 (b).

**Table 3:** Effect of each predictor on cumulative live birth rate of ART treatment adjusted for patient characteristics and stimulation information (Post-stimulation model)

Predictors	Coefficient	Odds ratio	95% CI	*P* value
Intercept	-2.615	0.073	0.042 ~ 0.129	< 0.001
Female age	0.045	1.046	1.028 ~ 1.065	< 0.001
Female age^a^	-0.217	0.805	0.783 ~ 0.828	< 0.001
No. of oocytes retrieved	0.254	1.289	1.243 ~ 1.338	< 0.001
No. of oocytes retrieved^a^	-0.362	0.696	0.614 ~ 0.789	< 0.001
No. of oocytes retrieved^b^	0.683	1.980	1.417 ~ 2.774	< 0.001
No. of previous IVF attempts, (vs. 0)				
1	-0.671	0.511	0.445 ~ 0.587	< 0.001
2	-0.837	0.433	0.342 ~ 0.547	< 0.001
> 2	-0.911	0.402	0.284 ~ 0.564	< 0.001
No. of previous ET failure, (vs. 0)				
1	0.333	1.396	1.182 ~ 1.649	< 0.001
2	0.523	1.686	1.327 ~ 2.146	< 0.001
> 2	0.711	2.036	1.421 ~ 2.921	< 0.001
Type of infertility, primary infertility vs. secondary infertility	0.156	1.168	1.081 ~ 1.263	< 0.001
Scarred uterus, (yes vs. no)	-0.189	0.828	0.736 ~ 0.932	0.002
Stimulation protocol, (vs. Follicular phase GnRH agonist protocol)				
Luteal phase GnRH agonist protocol	-0.362	0.696	0.609 ~ 0.797	< 0.001
GnRH antagonist protocol	-0.283	0.753	0.650 ~ 0.874	< 0.001
Others	-0.501	0.606	0.501 ~ 0.732	< 0.001
Endometrial thickness on trigger day, (≥ 7 mm vs. < 7 mm)	0.303	1.354	1.152 ~ 1.592	< 0.001
P level on trigger day, (vs. < 0.47)				
0.47 ~ 0.68	-0.008	0.992	0.889 ~ 1.108	0.890
0.69 ~ 0.97	-0.057	0.945	0.849 ~ 1.051	0.295
> 0.97	-0.205	0.814	0.731 ~ 0.907	< 0.001
LH level on trigger day, (vs. < 0.65)				
0.65 ~ 1.06	0.047	1.048	0.951 ~ 1.156	0.344
1.07 ~ 1.96	0.127	1.135	1.029 ~ 1.252	0.011
> 1.96	0.211	1.235	1.099 ~ 1.389	< 0.001

*IVF* In vitro fertilization, *ET* Embryo transfer, *GnRH* Gonadotropin-releasing hormone, *P* Progesterone, *LH* Luteinizing hormone

^a^The binomial of restricted cubic splines

^b^The trinomial of restricted cubic splines

### Prediction of CLBR by post-treatment model

To predict the CLBR in women who obtained implantable embryos, we remained 16,301 eligible cycles (embryo number > 0) and developed the post-treatment model after taking fertilization method and cumulative Day-3 embryos live-birth capacity as new predictors. In univariable analysis, all factors between live birth group and no live birth group had statistically significant differences, except for tubal infertility and intrauterine adhesion (Supplemental Table 3). After stepwise multivariable logistic regression modeling based on AIC criteria, the following 9 predictors were selected to explain the variation of cumulative live birth probabilities, as seen in Table 4. As shown in Fig. 2 (g, h), there were nonlinear relationships between the Day 3 embryo cumulative live-birth capacity and the ORs of CLBR with a J shaped curve. The odds began to be positive from the capacity of 1.036 (OR: 1.044, 95%CI: 1.002–1.087), and then rose exponentially. As the adjustment variable involved, the positive effect point moved forward to 0.776 (OR: 1.158, 95%CI: 1.026–1.308), and then the rising curve was elevated overall. The area under the ROC curve of post-treatment model was 0.8270 (Fig. 3, post-treatment model), which calibrated well (*p* = 0.549, Hosmer-Lemeshow test). The calibration curve was shown in Fig. 4 (c).

**Table 4:** Effect of each predictor on cumulative live birth rate of ART treatment adjusted for patient characteristics, stimulation and transferred embryo information (Post-treatment model)

Predictor	Coefficient	Odds ratio	95% CI	*P* value
Intercept	-2.014	0.134	0.072~0.249	<0.001
Female age	0.030	1.031	1.011~1.051	0.003
Female age^a^	-0.223	0.800	0.776~0.825	<0.001
No. of oocytes retrieved	0.005	0.995	0.959~1.033	0.808
No. of oocytes retrieved^a^	-0.091	0.913	0.801~1.039	0.166
No. of oocytes retrieved^b^	0.262	1.300	0.866~1.957	0.207
Cumulative Day3 embryos live-birth capacity	1.663	5.275	4.709~5.912	<0.001
Cumulative Day3 embryos live-birth capacity^a^	-1.039	0.354	0.297~0.423	<0.001
No. of previous IVF attempts, (vs 0)				
1	-0.240	0.787	0.708~0.874	<0.001
2	-0.154	0.857	0.699~1.050	0.138
>2	-0.317	0.729	0.528~0.995	0.049
Scarred uterus, (yes vs. no)	-0.200	0.818	0.721~0.929	0.002
Stimulation protocol, (Follicular phase GnRH agonist protocol vs.)				
Luteal phase GnRH agonist protocol	-0.288	0.750	0.645~0.872	0.001
GnRH antagonist protocol	-0.333	0.717	0.610~0.843	0.001
Others	-0.418	0.658	0.537~0.806	0.001
Endometrial thickness on trigger day, (≥7 mm vs. <7 mm)	0.401	1.493	1.254~1.779	<0.001
P level on trigger day, (vs <0.47)				
0.47~0.68	-0.039	0.962	0.852~1.085	0.524
0.69~0.97	-0.056	0.946	0.841~1.063	0.352
>0.97	-0.185	0.831	0.738~0.936	0.002
LH level on trigger day, (vs <0.65)				
0.65~1.06	-0.030	0.970	0.871~1.081	0.583
1.07~1.96	0.054	1.056	0.946~1.178	0.331
>1.96	0.177	1.194	1.051~1.358	0.007

*IVF* In vitro fertilization, *GnRH* Gonadotropin-releasing hormone, *P* Progesterone, *LH* Luteinizing hor-mone

^a^The binomial of restricted cubic splines

^b^The trinomial of restricted cubic splines

### Validation of CLBR prediction models

Independently, we used the internal validation set from the same source as the modeling data and temporal validation set from the different sources to evaluate these predictive models. As shown in Table 5, the C index of internal validation set were 0.7422 (0.7308 to 0.7536), 0.7722 (0.7612 to 0.7832), 0.8234 (0.8134 to 0.8333), respectively; and the calibration *P* values were all greater than 0.05, suggesting the relative robustness of models and no overfitting of predictor effects. In temporal validation, the C index were 0.7430 (0.7308 to 0.7536), 0.7722 (0.7612 to 0.7832), 0.8234 (0.8134 to 0.8333) respectively, suggesting that model prediction had a great repeatability; however, the calibration P values were less than 0.05.

**Table 5:** Internal and temporal validation of the predictive models

	C index	95% CI	Calibration c2	P value
**Baseline characteristics**				
Pre-treatment	0.7422	0.7308~0.7536	8.730	0.366
Post-stimulation	0.7722	0.7612~0.7832	10.151	0.255
Post-treatment	0.8234	0.8134~0.8333	12.337	0.137
Temporal validation				
Pre-treatment	0.7430	0.7307~0.7553	21.402	0.006
Post-stimulation	0.7747	0.7630~0.7863	83.618	<0.001
Post-treatment	0.8221	0.8113~0.8328	114.59	<0.001

### Visualization of CLBR prediction models

Finally, we developed an on-line calculator, in which clinicians and couples can use it to calculate their own CLBR (available on https://h5.eheren.com/hcyc/pc/index.html#/home). Detailed calculation formulas for three predictive models were showed in Supplemental text 2.

## Discussion

### Principal findings

We have developed a package of predictive models to estimate the individual CLBR at three different stages of treatment over one IVF cycle. We applied restricted cubic splines to explore the nonlinear effect between several predictors and the CLBR. In modeling, we found that predictors such as female age, AFC, number of oocytes retrieved and cumulative Day-3 embryos live-birth capacity had nonlinear correlations with live birth in different treatment stages. We have obtained more precisive prediction when the significant nonlinear terms were put into models.

### Interpretation of study findings and comparison with existing literature

These final models show that maternal age is a key factor in CLBR prediction during IVF treatment, no matter which stage the prediction was made. This conclusion was identical with previous studies [3, 9, 11, 12, 28]. In modeling, we recommend adding female age as a continuous variable to the prediction, which might clearly reflect the continuous effect of age change on personal live birth probability. Previous studies have reported the nonlinear relations between female age and pregnancy outcome with different methods. McLernon et al. found the nonlinear relation between the age and the probability of live birth using RCS in univariate analysis [4]. A study of Chen et al. also indicated that female age was nonlinearly associated with outcomes using generalized additive model [29]. And then they utilized the nonlinear relations to determine the cutoffs and segment the age in the process of modelling. However, this categorization approach was considered to be unreasonable because the specified truncation points in different studies was not uniform; the information of the variables was compressed into linear; and categorization assumed that the relationship between the predictor and the response is flat within intervals which was lack of consistency in most cases [26]. We therefore included the age with restricted cubic spline into multiple predications. Results showed that the accuracy of each model has been improved after considering nonlinear effect, and the effect persisted after adjustment for confounding variables at each stage, with an inverted S-shape curve.

AFC and the number of oocytes retrieved are also recognized as key predictors. It has been reported that basal AFC is able to predict live birth before receiving IVF treatment [17], and increasing AFC has a nonlinear association with higher odds of live birth [16, 17]. After considering the nonlinear effect of AFC with RCS and adding to prediction, we found that our pre-treatment model provided a higher precision than previous studies (C index = 0.7559). After including stimulation information, the association between AFC and CLBR became insignificant in the post-stimulation model, and instead, the oocytes number provided more significant effect. This might be due to the strong correlation between AFC and the number of oocytes retrieved. The conclusion that oocyte number has a nonlinear relation with pregnancy outcome has been reported already, with the rise in the odds of live birth gradually being flat when oocytes retrieved constantly increased [15, 30]. Therefore, our post-stimulation model (C index = 0.7744) has a similar CLBR prediction value to the pre-treatment model.

The number and quality of embryos transferred are considered to be key predictors for IVF/ICSI outcome. Terriou et al. found that embryo score predicted pregnancy better than the number of transferred embryos or female age [31]. Embryo morphology grading system has developed to be an international standard method to assess embryo quality in clinical practice [32]. However, although embryo score has been proved to be the independent predictor of live birth [11], few studies have taken embryo quality into account to predict CLBR as the number and quality of embryos couples obtained from one stimulation cycle are varied [3, 5]. Our study tentatively considered intact cleavage embryo information and quantified it by establishing LB regressions and then explored its prediction function for CLBR. We found the nonlinear association between cumulative embryo probabilities and odds of live birth, which indicated that the increase of the cumulative embryo quality brings a steep curve of CLBR. Ultimately, we obtained a more precisive predictive model for IVF/ICSI treatment (C index = 0.827).

### Clinical and research implications

We have built a set of predictive models to meet the needs of couples at different stages during IVF/ICSI treatment. These models are convenient and practical because information on all variables included was generally available clinically. In practice, if couples are successful at a certain stage, the following models will then provide revised predictions of CLBR according to information on this stage. These models might serve as a counseling tool in clinics. Couples could assess their own chances of delivery in each treatment stage according to personalized conditions, and clinicians could adjust reliable treatment protocols according to couples’ basic characteristics. The results from our model might help couples plan their time and prepare emotionally and financially for their complete IVF journey.

In prior studies, models were built based on national data [3, 4], but these data often came from multiple clinics, leading to variance of IVF/ICSI techniques and diagnostic criteria, as well as the absence of some potentially important predictors, such as P level on trigger day. Our single-center study confirmed the prediction value of several clinical indicators for CLBR, which have been reported previously [12, 33–36]. To the best of our knowledge, the C index of our models are the highest for ART models to date. This might be due to the inclusion of non-linearity, the effect of complete embryo information, or a combination of both.

### Strengths and limitations

The present study is strengthened by the inclusion of over 30,000 treatment cycles, the use of RSC for model construction, and the consideration of CLBR over a complete cycle as the study subject. Our detailed medical data, including fertility check-up, stimulation characteristics and embryo outcomes, allowed us to develop a package of models at different stages of treatment with high precision. In addition, both internal validation and temporal validation were applied to verify the robustness and good discrimination of the predictive models.

Whereas our models showed good results in the test sets, the current study contains several potential limitations. First, some factors associated with men were not considered in our models, such as male age and BMI. However, previous studies have reported that male BMI did not influence both LBR and CLBR [37, 38], and one study pointed that paternal age had no association with LBR after adjusting for female age [39]. Nonetheless, some potentially important predictors were poorly recorded and should be better considered in future study, such as ethnicity and AMH level. Second, although we examined our models with internal population in internal and temporal validation, it was lack of external validation on independent data, and its generalizability needs to be confirmed. Moreover, the temporal validation showed that the Hosmer-Lemeshow test χ^2^ in the calibration were relatively large, implying low accuracy of the predictive models possibly attributed to time-associated amelioration of IVF practice. Third, this study was conducted in a single center. The clinical application and generalizability of the findings are restricted due to the specific characteristics of the study population and regional variations in IVF practices. To expand the applicable population for the predictive models, further multicenter studies are needed in the future.

## Conclusions

In summary, we have built a set of IVF/ICSI models to predict CLBR according to information from different treatment stages. In modeling, we included the effect of non-linearity of several key predictors, such as female age, basal AFC, oocyte number and cumulative embryo live birth probabilities. Our models show good stability and should provide a practically useful consulting tool for both couples and clinicians.

### Supplementary Information

Supplementary Material 1.

Supplementary Material 2.

Supplementary Material 3.

Supplementary Material 4.

Supplementary Material 5.

## Data Availability

No datasets were generated or analysed during the current study.

## Abbreviations

CLBR: cumulative live birth rate
IVF: in vitro fertilization
ET: embryo transfer
ART: assisted reproductive technology
AFC: assisted reproductive technology
ROC: receiver operating characteristic
FET: frozen embryo transfer
ICSI: intracytoplasmic sperm injection
TVUS: transvaginal ultrasound
GnRH: gonadotropin-releasing hormone
hCG: human chorionic gonadotrophin
SD: standard deviation
RCS: restricted cubic spline
BMI: body mass index
E2: estradiol
P: progesterone
LH: luteinizing hormone
AIC: akaike information criteria
OR: odds ratio
