# Supplementary material for: Predicting personalized cumulative live birth rate after a complete in vitro fertilization cycle: an analysis of 32,306 treatment cycles in China
Source: Reprod Biol Endocrinol. 2024 Jun 7;22:65. doi: 10.1186/s12958-024-01237-3 (PMC11158004; doi:10.1186/s12958-024-01237-3)
Supplement: Supplementary file 1 — Supplementary Material 1. [file 12958_2024_1237_MOESM1_ESM.docx]

**Supplemental Table 1 Univariable analysis of CLBR in the pre-treatment stage**

| Characteristics | Non-live birth  (n = 7555) | Live birth  (n = 10367) | P value |
| --- | --- | --- | --- |
| Female age (y), Median (interquartile range) | 34 (10) | 29 (7) | <0.001 |
| Antral follicles count, Median (interquartile range) | 8 (7) | 13 (8) | <0.001 |
| Female BMI (kg/m^2^), n (%) |  |  | <0.001 |
| <18.5 | 683 (9.04) | 1139 (10.99) |  |
| 18.5~23.9 | 5053(66.88) | 6989 (67.42) |  |
| 24.0~28.0 | 1511 (20.00) | 1856 (17.90) |  |
| >28.0 | 308 (4.08) | 383 (3.69) |  |
| Duration of infertility (y), n (%) |  |  | <0.001 |
| <2 | 2261 (29.93) | 3394 (32.74) |  |
| 2~5 | 2721 (36.02) | 4398 (42.42) |  |
| >5 | 2573 (34.06) | 2575 (24.84) |  |
| No. of abortion, n (%) |  |  | <0.001 |
| 0 | 4477 (59.26) | 6802 (65.61) |  |
| 1 | 1816 (24.04) | 2438 (23.52) |  |
| 2 | 743 (9.83) | 768 (7.41) |  |
| >2 | 519 (6.87) | 359 (3.46) |  |
| No. of previous IVF attempts,n (%) |  |  | <0.001 |
| 0 | 4877 (64.55) | 8821 (85.09) |  |
| 1 | 1759 (23.28) | 1253 (12.09) |  |
| 2 | 543 (7.19) | 227 (2.19) |  |
| >2 | 376 (4.98) | 66 (0.64) |  |
| No. of previous ET failure, n (%) |  |  | <0.001 |
| 0 | 6040 (79.95) | 9245 (89.18) |  |
| 1 | 1085 (14.36) | 777 (7.49) |  |
| 2 | 301 (3.98) | 252 (2.43) |  |
| >2 | 129 (1.71) | 93 (0.90) |  |
| Type of infertility, n (%) |  |  | <0.001 |
| Primary infertility | 2985 (39.51) | 4768 (45.99) |  |
| Secondary infertility | 4570 (60.49) | 5599 (54.01) |  |
| Infertility diagnosis, n (%) |  |  |  |
| Tubal factor | 5356 (70.89) | 7546 (72.79) | 0.005 |
| Male factor | 1936 (25.63) | 2956 (28.51) | <0.001 |
| Ovulatory disorder | 750 (9.93) | 1757 (16.95) | <0.001 |
| Endometriosis | 566 (7.49) | 650 (6.27) | 0.001 |
| PCOS | 385 (5.10) | 1190 (11.48) | <0.001 |
| Intrauterine adhesion | 1094 (14.48) | 1589 (15.33) | 0.117 |
| Scarred uterus | 1021 (13.51) | 863 (8.32) | <0.001 |
| *BMI,* body mass index; *IVF,* in vitro fertilization; *ET,* embryo transfer; *PCOS,* polycystic ovary syndrome. | | | |
