# Supplementary material for: Predicting personalized cumulative live birth rate after a complete in vitro fertilization cycle: an analysis of 32,306 treatment cycles in China
Source: Reprod Biol Endocrinol. 2024 Jun 7;22:65. doi: 10.1186/s12958-024-01237-3 (PMC11158004; doi:10.1186/s12958-024-01237-3)
Supplement: Supplementary file 2 — Supplementary Material 2. [file 12958_2024_1237_MOESM2_ESM.docx]

**Supplemental Table 2 Univariable analysis of CLBR in the post-stimulation stage**

| Characteristics | Non-live birth  (n = 7057) | Live birth  (n = 10366) | P value |
| --- | --- | --- | --- |
| **Baseline characteristics** |  |  |  |
| Female age (y), Median (interquartile range) | 33 (10) | 29 (7) | <0.001 |
| Antral follicles count, Median (interquartile range) | 8 (8) | 13 (8) | <0.001 |
| Female BMI (kg/m^2^), n (%) |  |  | <0.001 |
| <18.5 | 645 (9.14) | 1142 (11.02) |  |
| 18.5~23.9 | 4727 (66.98) | 7007 (67.60) |  |
| 24.0~28.0 | 1428 (20.24) | 1845 (17.80) |  |
| >28.0 | 257 (3.64) | 372 (3.59) |  |
| Duration of infertility (y), n (%) |  |  | <0.001 |
| <2 | 2101 (29.77) | 3344 (32.26) |  |
| 2~5 | 2602 (36.87) | 4443 (42.86) |  |
| >5 | 2354 (33.36) | 2579 (24.88) |  |
| No. of abortion, n (%) |  |  | <0.001 |
| 0 | 4199 (59.50) | 6758 (65.19) |  |
| 1 | 1675 (23.74) | 2428 (23.42) |  |
| 2 | 707 (10.02) | 803 (7.75) |  |
| >2 | 476 (6.75) | 377 (3.64) |  |
| No. of previous IVF attempts, n (%) |  |  | <0.001 |
| 0 | 4658 (66.01) | 8796 (84.85) |  |
| 1 | 1614 (22.87) | 1277 (12.32) |  |
| 2 | 490 (6.94) | 219 (2.11) |  |
| >2 | 295 (4.18) | 74 (0.71) |  |
| No. of previous ET failure, n (%) |  |  | <0.001 |
| 0 | 5644 (79.98) | 9225 (88.99) |  |
| 1 | 996 (14.11) | 784 (7.56) |  |
| 2 | 293 (4.15) | 260 (2.51) |  |
| >2 | 124 (1.76) | 97 (0.94) |  |
| Type of infertility, n (%) |  |  | <0.001 |
| Primary infertility | 2818 (39.93) | 4726 (45.59) |  |
| Secondary infertility | 4239 (60.07) | 5640 (54.41) |  |
| Infertility diagnosis, n (%) |  |  |  |
| Tubal factor | 5041 (71.43) | 7529 (72.63) | 0.083 |
| Male factor | 1857 (26.31) | 2945 (28.41) | 0.002 |
| Ovulatory disorder | 700 (9.92) | 1717 (16.56) | <0.001 |
| Endometriosis | 542 (7.68) | 670 (6.46) | 0.002 |
| PCOS | 360 (5.10) | 1173 (11.32) | <0.001 |
| Intrauterine adhesion | 1023 (14.50) | 1561 (15.06) | 0.305 |
| Scarred uterus | 922 (13.07) | 898 (8.66) | <0.001 |
| **Ovarian stimulation characteristics** |  |  |  |
| Stimulation protocol, n (%) |  |  | <0.001 |
| follicular phase GnRH agonists protocol | 4230 (59.94) | 8835 (85.23) |  |
| luteal phase GnRH agonists protocol | 480 (6.80) | 756 (7.29) |  |
| GnRH antagonist protocol | 888 (12.58) | 492 (4.75) |  |
| Others | 1459 (20.67) | 283 (2.73) |  |
| Endometrial thickness on trigger day (mm), n (%) |  |  | <0.001 |
| <7 | 857 (12.14) | 359 (3.46) |  |
| ≥7 | 6200 (87.86) | 10007 (96.54) |  |
| E2 level on trigger day (pg/mL), n (%) |  |  | <0.001 |
| <1049 | 2596 (36.79) | 1087 (10.49) |  |
| 1049~1796 | 1853 (26.26) | 2607 (25.15) |  |
| 1796~2751 | 1466 (20.77) | 3053 (29.45) |  |
| >2751 | 1142 (16.18) | 3619 (34.91) |  |
| P level on trigger day (ng/mL), n (%) |  |  | <0.001 |
| <0.47 | 1739 (24.64) | 1683 (16.24) |  |
| 0.47~0.69 | 1751 (24.81) | 2201 (21.23) |  |
| 0.69~0.97 | 1723 (24.42) | 3088 (29.79) |  |
| >0.97 | 1844 (26.13) | 3394 (32.74) |  |
| LH level on trigger day (IU/L), n (%) |  |  | <0.001 |
| <0.65 | 1486 (21.06) | 2855 (27.54) |  |
| 0.65~1.07 | 1442 (20.43) | 2735 (26.38) |  |
| 1.07~1.96 | 1693 (23.99) | 2929 (28.26) |  |
| >1.96 | 2436 (34.52) | 1847 (17.82) |  |
| Types of trigger, n (%) |  |  | <0.001 |
| hCG | 5731 (81.21) | 10094 (97.38) |  |
| GnRH agonist | 908 (12.87) | 165 (1.59) |  |
| hCG + GnRH agonist | 418 (5.92) | 107 (1.03) |  |
| No. of oocytes retrieved, Median (interquartile range) | 7 (8) | 12 (8) | <0.001 |
| *BMI,* body mass index; *IVF,* in vitro fertilization; *ET,* embryo transfer; *PCOS,* polycystic ovary syndrome; *GnRH,* gonadotropin-releasing hormone; *E2,* estradiol; *P,* progesterone; *LH,* luteinizing hormone; *hCG*, human chorionic gonadotrophin. | | | |
