# Supplementary material for: Predicting personalized cumulative live birth rate after a complete in vitro fertilization cycle: an analysis of 32,306 treatment cycles in China
Source: Reprod Biol Endocrinol. 2024 Jun 7;22:65. doi: 10.1186/s12958-024-01237-3 (PMC11158004; doi:10.1186/s12958-024-01237-3)
Supplement: Supplementary file 3 — Supplementary Material 3. [file 12958_2024_1237_MOESM3_ESM.docx]

**Supplemental Table 3 Univariable analysis of CLBR in the post-treatment stage**

|  | Non-live birth  (n = 5941) | Live birth  (n = 10360) | P value |
| --- | --- | --- | --- |
| **Baseline characteristics** |  |  |  |
| Female age (y), Median (interquartile range) | 33 (10) | 29 (7) | <0.001 |
| Antral follicles count, Median (interquartile range) | 9 (8) | 13 (8) | <0.001 |
| Female BMI (kg/m^2^), n (%) |  |  | <0.001 |
| <18.5 | 558 (9.39) | 1149(11.09) |  |
| 18.5~23.9 | 3952 (66.52) | 6993(67.50) |  |
| 24.0~28.0 | 1193 (20.08) | 1833(17.69) |  |
| >28.0 | 238 (4.01) | 385(3.72) |  |
| Duration of infertility (y), n (%) |  |  | <0.001 |
| <2 | 1811 (30.48) | 3381 (32.64) |  |
| 2~5 | 2165 (36.44) | 4424 (42.70) |  |
| >5 | 1965 (33.08) | 2555 (24.66) |  |
| No. of abortion, n (%) |  |  | <0.001 |
| 0 | 3492 (58.78) | 6791 (65.55) |  |
| 1 | 1414 (23.80) | 2404 (23.20) |  |
| 2 | 612 (10.30) | 790 (7.63) |  |
| >2 | 423 (7.12) | 375 (3.62) |  |
| No. of previous IVF attempts,n (%) |  |  | <0.001 |
| 0 | 4053 (68.22) | 8795 (84.89) |  |
| 1 | 1317 (22.17) | 1269 (12.25) |  |
| 2 | 368 (6.19) | 227 (2.19) |  |
| >2 | 203 (3.42) | 69 (0.67) |  |
| No. of previous ET failure, n (%) |  |  | <0.001 |
| 0 | 4053 (68.22) | 8795 (84.89) |  |
| 1 | 1317 (22.17) | 1269 (12.25) |  |
| 2 | 368 (6.19) | 227 (2.19) |  |
| >2 | 203 (3.42) | 69 (0.67) |  |
| Type of infertility, n (%) |  |  | <0.001 |
| Primary infertility | 2302 (38.75) | 4722 (45.58) |  |
| Secondary infertility | 3639 (61.25) | 5638 (54.42) |  |
| Infertility diagnosis, n (%) |  |  |  |
| Tubal factor | 4311 (72.56) | 7537 (72.75) | 0.796 |
| Male factor | 1538 (25.89) | 2875 (27.75) | 0.01 |
| Ovulatory disorder | 598 (10.07) | 1739 (16.79) | <0.001 |
| Endometriosis | 443 (7.46) | 670 (6.47) | 0.016 |
| PCOS | 349 (5.87) | 1186 (11.45) | <0.001 |
| Intrauterine adhesion | 889 (14.96) | 1625 (15.69) | 0.220 |
| Scarred uterus | 794 (13.36) | 876 (8.46) | <0.001 |
| **Ovarian stimulation characteristics** |  |  |  |
| Stimulation protocol, n (%) |  |  | <0.001 |
| follicular phase GnRH agonists protocol | 3869 (65.12) | 8805 (84.99) |  |
| luteal phase GnRH agonists protocol | 421 (7.09) | 790 (7.63) |  |
| GnRH antagonist protocol | 726 (12.22) | 493 (4.76) |  |
| Others | 925 (15.57) | 272 (2.63) |  |
| Endometrial thickness on trigger day (mm), n (%) |  |  | <0.001 |
| <7 | 654 (11.01) | 352 (3.40) |  |
| ≥7 | 5287 (88.99) | 10008 (96.60) |  |
| E2 level on trigger day (pg/mL), n (%) |  |  | <0.001 |
| <1049 | 1831 (30.82) | 1066 (10.29) |  |
| 1049~1796 | 1678 (28.24) | 2565 (24.76) |  |
| 1796~2751 | 1342 (22.59) | 3114 (30.06) |  |
| >2751 | 1090 (18.35) | 3615 (34.89) |  |
| P level on trigger day (ng/mL), n (%) |  |  | <0.001 |
| <0.47 | 1396 (23.50) | 1701 (16.42) |  |
| 0.47~0.69 | 1406 (23.67) | 2178 (21.02) |  |
| 0.69~0.97 | 1554 (26.16) | 3122 (30.14) |  |
| >0.97 | 1585 (26.68) | 3359 (32.42) |  |
| LH level on trigger day (IU/L), n (%) |  |  | <0.001 |
| <0.65 | 1360 (22.89) | 2884 (27.84) |  |
| 0.65~1.07 | 1331 (22.40) | 2713 (26.19) |  |
| 1.07~1.96 | 1441 (24.26) | 2911 (28.10) |  |
| >1.96 | 1809 (30.45) | 1852 (17.88) |  |
| Types of trigger, n (%) |  |  | <0.001 |
| hCG | 5065 (85.26) | 10099 (97.48) |  |
| GnRH agonist | 565 (9.51) | 154 (1.49) |  |
| hCG + GnRH agonist | 311 (5.23) | 107 (1.03) |  |
| No. of oocytes retrieved, Median (interquartile range) | 8 (7) | 12 (8) | <0.001 |
| **Embryo transfer characteristics** |  |  |  |
| Artificial insemination technology, n (%) |  |  | <0.001 |
| IVF | 4243 (71.42) | 7868 (75.95) |  |
| ICSI | 1435 (24.15) | 1990 (19.21) |  |
| IVF + ICSI | 263 (4.43) | 502 (4.85) |  |
| Cumulative Day-3 embryos live birth capacity, Median (interquartile range) | 0.85(1.00) | 2.01(1.73) | <0.001 |
| *BMI,* body mass index; *IVF,* in vitro fertilization; *ET,* embryo transfer; *PCOS,* polycystic ovary syndrome; *GnRH,* gonadotropin-releasing hormone; *E2,* estradiol; *P,* progesterone; *LH,* luteinizing hormone; *hCG*, human chorionic gonadotrophin; *ICSI,* intracytoplasmic sperm injection. | | | |
