# Supplementary material for: Predicting personalized cumulative live birth rate after a complete in vitro fertilization cycle: an analysis of 32,306 treatment cycles in China
Source: Reprod Biol Endocrinol. 2024 Jun 7;22:65. doi: 10.1186/s12958-024-01237-3 (PMC11158004; doi:10.1186/s12958-024-01237-3)
Supplement: Supplementary file 4 — Supplementary Material 4. [file 12958_2024_1237_MOESM4_ESM.docx]

**Supplemental text 1 The model information and calculation formula for cumulative Day3 embryos live-birth capacity**

**Parameter estimates**

| Predictor | Coefficient | Odds ratio | 95% CI | P value |
| --- | --- | --- | --- | --- |
| Intercept | -2.872 |  |  | <0.001 |
| Rating of cell number | 0.214 | 1.238 | 1.143~1.341 | <0.001 |
| Rating of fragmentation | 0.121 | 1.129 | 1.007~1.265 | 0.037 |
| Symmetry | 0.547 | 1.727 | 1.335~2.234 | <0.001 |

**Variable information**

Rating of cell number: 1 = “5 cell”; 2 = “6 cell”; 3 = “7 cell”; 4 = “9 cell”; 5 = “8 cell”; 6 = “10-11 cell”; 7 = “12-13 cell”; 8 = “≥14 cell”.

Rating of fragmentation: 1 = “>40%”; 2 = “36-40%”; 3 = “31-35%”; 4 = “26-30%”; 5 = “21-25%”; 6 = “16-20%”; 7 = “10-15%”; 8 = “<10%”.

Symmetry: 1 = “even”; 2 = “uneven”.

**Equation**

P=1-1/(1+exp(-2.872+0.214*Rating of cell number+0.121*Rating of fragmentation+0.547*Symmetry

**Calculation of cumulative Day3 embryos live-birth capacity**

Assume that there were *n* Day3 implantable embryos in a complete cycle, and calculate the probability of live birth for each embryo (P_1_, P_2_, ... P_n_), and then added them up by cycle (P_1_+P_2_...+P_n_).
