# Supplementary material for: Predicting personalized cumulative live birth rate after a complete in vitro fertilization cycle: an analysis of 32,306 treatment cycles in China
Source: Reprod Biol Endocrinol. 2024 Jun 7;22:65. doi: 10.1186/s12958-024-01237-3 (PMC11158004; doi:10.1186/s12958-024-01237-3)
Supplement: Supplementary file 5 — Supplementary Material 5. [file 12958_2024_1237_MOESM5_ESM.docx]

**Supplemental text 2 Calculation formula for cumulative live birth rate of three predictive models**

**Variable information**

Continuous variable: Female age, Antral follicle count, No. of oocytes retrieved, Cumulative Day-3 embryos live birth capacity.

Categorical variable: Scarred uterus, Female BMI, Type of infertility, Tubal factor, Male factor, No. of previous IVF attempts, No. of previous ET failure, Stimulation protocol, Endometrial thickness on trigger day, Progesterone level on trigger day, Luteinizing hormone level on trigger day.

Scarred uterus: 1 = “yes”; 0 = “no”.

Female BMI (kg/m2): 1 = “<18.5”; 2 = “18.5~23.9”; 3 = “24.0~27.9”; 4 = “≥28.0”.

Type of infertility: 1 = “secondary infertility”; 0 = “primary infertility”.

Tubal factor: 1 = “yes”; 0 = “no”.

Male factor: 1 = “yes”; 0 = “no”.

No. of previous IVF attempts: 0 = “0”; 1 = “1”; 2 = “2”; 3 = “>2”.

No. of previous ET failure: 0 = “0”; 1 = “1”; 2 = “2”; 3 = “>2”.

Stimulation protocol: 1 = “Follicular phase GnRH agonists protocol”; 2 = “Luteal phase GnRH agonists protocol”; 3 = “GnRH antagonist protocol”; 4 = “Others”.

Endometrial thickness on trigger day (mm): 1 = “≥7”; 0 = “<7”.

Progesterone level on trigger day (ng/mL): 1 = “<0.47”; 2 = “0.47~0.68”; 3 = “0.69~0.97”; 4 = “>0.97”.

Luteinizing hormone level on trigger day (IU/L): 1 = “<0.65”; 2 = “0.65~1.06”; 3 = “1.07~1.96”; 4 = “>1.96”.

**Equation for pre-treatment model:**

P=1-1/(1+exp(-1.8257735

+0.042664062*Female age-0.0010521781*pmax(Female age-25,0)^3+0.0018413117*pmax(Female age-31,0)^3-0.00078913357*pmax(Female age-39,0)^3

+0.15758158*Antral follicle count-0.00040718107*pmax(Antral follicle count-3,0)^3+0.00041326983*pmax(Antral follicle count-8,0)^3+0.00017622594*pmax(Antral follicle count-13,0)^3-0.00018231469*pmax(Antral follicle count-24,0)^3

-0.21986734*(Scarred uterus=="1")

+0.065044611*(Female BMI=="2")-0.035468845*(Female BMI=="3")-0.21247987*(Female BMI=="4")

+0.14800763*(Type of infertility=="1")

+0.11879724*(Tubal factor=="1")

+0.13718847*(Male factor=="1")

-0.94391073*(No. of previous IVF attempts=="1")-1.2577584*(No. of previous IVF attempts=="2")-1.9363406*(No. of previous IVF attempts=="3")

+0.44858493*(No. of previous ET failure=="1")+0.8363917*(No. of previous ET failure=="2")+0.99158811*(No. of previous ET failure=="3"))

**Equation for post-stimulation model:**

P=1-1/(1+exp(-2.6145752

+0.045108255*Female age-0.001106532*pmax(Female age-25,0)^3+0.0017212719*pmax(Female age-30,0)^3-0.00061473997*pmax(Female age-39,0)^3

+0.25425422*No. of oocytes retrieved-0.00082070368*pmax(No. of oocytes retrieved-2,0)^3+0.0015493528*pmax(No. of oocytes retrieved-8,0)^3-0.0006005515*pmax(No. of oocytes retrieved-13,0)^3-0.00012809764*pmax(No. of oocytes retrieved-23,0)^3

-0.18856487*(Scarred uterus=="1")

+0.15562478*(Type of infertility=="1")

-0.67145556*(No. of previous IVF attempts=="1")-0.83727365*(No. of previous IVF attempts=="2")-0.91067522*(No. of previous IVF attempts=="3")

+0.33338393*(No. of previous ET failure=="1")+0.52253677*(No. of previous ET failure=="2")+0.71081355*(No. of previous ET failure=="3")

-0.36227133*(Stimulation protocol=="2")-0.28307208*(Stimulation protocol=="3")-0.50079315*(Stimulation protocol=="4")

+0.30309654*(Endometrial thickness on trigger day=="1")

-0.0077661583*(Progesterone level on trigger day=="2")-0.057090294*(Progesterone level on trigger day=="3")-0.20519869*(Progesterone level on trigger day=="4")

+0.047083857*(Luteinizing hormone level on trigger day=="2")+0.12664349*(Luteinizing hormone level on trigger day=="3")+0.21131893 *(Luteinizing hormone level on trigger day=="4"))

**Equation for post-treatment model:**

P=1-1/(1+exp(-2.0136506

+0.030296572*Female age-0.0011373963*pmax(Female age-25,0)^3+0.0017692831*pmax(Female age-30,0)^3-0.00063188682*pmax(Female age-39,0)^3

-0.004577405*No. of oocytes retrieved-0.00022869701*pmax(No. of oocytes retrieved-3,0)^3+0.00065565382*pmax(No. of oocytes retrieved-9,0)^3-0.00046052132*pmax(No. of oocytes retrieved-13,0)^3+3.3564517*10^-5*pmax(No. of oocytes retrieved-23,0)^3

+1.6629679*Cumulative Day-3 embryos live birth capacity-0.10157352*pmax(Cumulative Day-3 embryos live birth capacity-0.4050314,0)^3+0.15659058*pmax(Cumulative Day-3 embryos live birth capacity-1.52868,0)^3-0.055017058*pmax(Cumulative Day-3 embryos live birth capacity-3.603183,0)^3

-0.20049227*(Scarred uterus=="1")

-0.23963854*(No. of previous IVF attempts=="1")-0.15375331*(No. of previous IVF attempts=="2")-0.31655059*(No. of previous IVF attempts=="3")

-0.28787188*(Stimulation protocol=="2")-0.33269338*(Stimulation protocol=="3")-0.41808278*(Stimulation protocol=="4")

+0.40089012*(Endometrial thickness on trigger day=="1")

-0.039258271*(Progesterone level on trigger day=="2")-0.055532922*(Progesterone level on trigger day=="3")-0.18486345*(Progesterone level on trigger day=="4")

-0.030256109*(Luteinizing hormone level on trigger day=="2")+0.054309969*(Luteinizing hormone level on trigger day=="3")+0.17743229*(Luteinizing hormone level on trigger day=="4"))

**Function Interpretation**

Exp(x): e^x^

Pmax(x,y): Maximum value between x and y

X^3: Cubic of X

(X=="Y"): If X equals Y, output 1; otherwise, output 0
